# Reducing child abuse amongst adolescents in low- and middle-income countries: A pre-post trial in South Africa

**DOI:** 10.1186/s12889-016-3262-z

**Published:** 2016-07-13

**Authors:** Lucie Cluver, Franziska Meinck, Alexa Yakubovich, Jenny Doubt, Alice Redfern, Catherine Ward, Nasteha Salah, Sachin De Stone, Tshiamo Petersen, Phelisa Mpimpilashe, Rocio Herrero Romero, Lulu Ncobo, Jamie Lachman, Sibongile Tsoanyane, Yulia Shenderovich, Heidi Loening, Jasmina Byrne, Lorraine Sherr, Lauren Kaplan, Frances Gardner

**Affiliations:** 1 Department of Social Policy & Intervention, Centre for Evidence-Based Intervention, University of Oxford Barnett House, 32 Wellington Square, Oxford, OX1 2ER UK; 2 Department of Psychiatry and Mental Health, University of Cape Town, Cape Town, South Africa; 3 Department of Psychology, University of Cape Town, Cape Town, South Africa; 4 Clowns Without Borders South Africa, PO Box 18670, Durban, 4014 South Africa; 5 UNICEF, Offices of Research – Innocenti, Florence, Italy; 6 Royal Free and University College Medical School, University College London, London, UK; 7 Alcohol Research Group, Public Health Institute, Emeryville, California USA

**Keywords:** Child abuse, Prevention, Parenting, Abuse prevention, Parenting stress, Psycho-social aspects, South Africa

## Abstract

**Background:**

No known studies have tested the effectiveness of child abuse prevention programmes for adolescents in low- or middle-income countries. ‘Parenting for Lifelong Health’ (http://tiny.cc/whoPLH) is a collaborative project to develop and rigorously test abuse-prevention parenting programmes for free use in low-resource contexts. Research aims of this first pre-post trial in South Africa were: i) to identify indicative effects of the programme on child abuse and related outcomes; ii) to investigate programme safety for testing in a future randomised trial, and iii) to identify potential adaptations.

**Methods:**

Two hundred thirty participants (adolescents and their primary caregivers) were recruited from schools, welfare services and community-sampling in rural, high-poverty South Africa (no exclusion criteria). All participated in a 12-week parenting programme, implemented by local NGO childcare workers to ensure real-world external validity. Standardised pre-post measures with adolescents and caregivers were used, and paired t-tests were conducted for primary outcomes: abuse (physical, emotional abuse and neglect), adolescent behaviour problems and parenting (positive and involved parenting, poor monitoring and inconsistent discipline), and secondary outcomes: mental health, social support and substance use.

**Results:**

Participants reported high levels of socio-economic deprivation, e.g. 60 % of adolescents had either an HIV-positive caregiver or were orphaned by AIDS, and 50 % of caregivers experienced intimate partner violence. i) indicative effects: Primary outcomes comparing pre-test and post-test assessments showed reductions reported by adolescents and caregivers in child abuse (adolescent report 63.0 % pre-test to 29.5 % post-test, caregiver report 75.5 % pre-test to 36.5 % post-test, both *p* < 0.001) poor monitoring/inconsistent discipline (*p* < .001), adolescent delinquency/aggressive behaviour (both *p* < .001), and improvements in positive/involved parenting (*p* < .01 adolescent report, *p* < .001 caregiver report). Secondary outcomes showed improved social support (*p* < .001 adolescent and caregiver reports), reduced parental and adolescent depression (both *p* < .001), parenting stress (*p* < .001 caregiver report) and caregiver substance use (*p* < .002 caregiver report). There were no changes in adolescent substance use. No negative effects were detected. ii) Programme acceptability and attendance was high. There was unanticipated programme diffusion within some study villages, with families initiating parenting groups in churches, and diffusion through school assemblies and religious sermons. iii) potential adaptations identified included the need to strengthen components on adolescent substance use and to consider how to support spontaneous programme diffusion with fidelity.

**Conclusions:**

The programme showed no signs of harm and initial evidence of reductions in child abuse and improved caregiver and adolescent outcomes. It showed high acceptability and unexpected community-level diffusion. Findings indicate needs for adaptations, and suitability for the next research step of more rigorous testing in randomised trials, using cluster randomization to allow for diffusion effects.

## Background

Adolescents in low- and middle-income countries (LMIC) such as South Africa bear a disproportionate burden of abuse and violence [1, 2]. Globally, an estimated 95,000,000 children experience abuse annually, with the highest rates in the WHO Africa region [1]. In 2015, the first representative study in South Africa reported lifetime rates of 34 % physical abuse, 16 % emotional abuse and 20 % sexual abuse amongst 15–17 year olds [3]. Although the evidence-base is scattered, studies show that pathways of poverty, caregiver^1^ mental health distress and HIV/AIDS contribute to harsh parenting and maltreatment [2]. There is clear evidence that exposure to abuse has severe and lasting adverse effects on adolescents’ health, education, employment, mortality and subsequent risk of HIV-infection [4, 5]. In the context of Africa’s population ‘youth bulge’ [6] and emerging evidence of continued brain development - neural, functional and emotional - during adolescence [7], it is increasingly clear that later childhood development is an important time for intervention.

In 2009, a systematic review of reviews identified parenting programmes as having a promising evidence-base for child abuse prevention [8], but also found a lack of research from LMIC. There is a small evidence-base in LMIC for parenting programmes targeted at younger children, with two emerging studies for children under 10 years-old in South Africa [9] and Liberia [10]. For adolescents, the evidence-base is severely lacking: to date there are no known published studies of parenting programmes for adolescent abuse prevention in LMIC [11].

A number of parenting programmes have shown effectiveness in child abuse prevention and reduction, and good transportability within high-income countries. These include The Incredible Years, Triple P, Parent–child Interaction Therapy (PCIT) and Parent Management Training Oregon (PMTO) (all for pre-teens) and the Nurse-Family Partnership (for pregnancy and infants) [12, 13]. All use social learning theory approaches, build parenting skills in behaviour management and promote family problem-solving. However, many existing evidence-based parenting programmes charge fees for training, manuals and equipment that make them prohibitive for low-resource contexts. Others are only able to be implemented by qualified nurses or psychologists - professions in extremely short supply in LMIC, and none are designed for adolescents.

In response to these challenges for LMIC, an international collaboration, ‘Parenting for Lifelong Health’ (http://tiny.cc/whoPLH) was initiated in 2012, with the World Health Organization (WHO), UNICEF, and academics from the global South and North. This collaboration is developing and rigorously testing a suite of child abuse prevention programmes for different child developmental stages [14], with support from many donor partners, LMIC governments and PEPFAR-USAID. Programmes are designed for low-resource contexts, are implemented by lay community workers in local NGOs or government services and require little or no equipment, nor availability of electricity. If found effective, programmes will become freely available under licensing that prohibits any profit-making or commercial interests.

This study concerns the adolescent programme. Development, testing and adaptation stages are iterative, using the Medical Research Council’s (MRC) model for intervention development [15]. First, systematic reviews were used to identify shared core components of effective parenting programmes [12], and the evidence for transportability of parenting programmes to low- and middle-income contexts [11, 13]. Second, draft manuals were co-developed by academics (Universities of Oxford and Cape Town) and NGO partners (Clowns Without Borders South Africa). Third, over fifty international academics, practitioners and programming experts provided comments and advice on initial drafts. Fourth, six months of in-depth qualitative research in South Africa identified adaptation needs such as including information on child rape, which is common in South Africa. Fifth, an initial pilot stage was conducted (*n* = 60 participants in rural South Africa), showing high levels of acceptability, pre-post test improvements in parent and adolescent outcomes, and no iatrogenic effects. Subsequently, the programme was adapted based on small-scale pre-post test results, observer-coding and qualitative feedback from participants and staff.

The current study comprises the next major step in programme testing: a larger pre-post trial in order to test indicative effects, programme acceptability and to identify further adaptation needs in real-world conditions. Programme implementation was delivered by childcare workers within King William’s Town Child and Youth Care Centre, part of Isibindi, a community-based programme founded by the National Association of Child and Youth Care Workers. These local staff were trained and supervised by another NGO, Clowns Without Borders South Africa, itself funded by the Provincial government’s Department of Social Development and UNICEF South Africa. The study took place in six rural and peri-urban communities in one of South Africa’s poorest provinces: the Eastern Cape. The area was selected by the Department of Social Development and UNICEF South Africa, as representative of the country’s most deprived and service-challenged contexts – it is characterized by high levels of HIV, unemployment, crime and by overburdened social and health services [16]. Low levels of infrastructure in rural areas include very limited roads, no electricity and frequent lack of water supplies: such a context is potentially more generalizable to other parts of Southern Africa than the major cities in which programmes are more frequently first tested. Programming and research took place in close partnership with the Provincial and National Departments of Social Development and Education and UNICEF. Aims of the study were: 1) to identify indicative effects of the programme on child abuse and related outcomes; 2) to investigate programme safety for testing in a future randomised controlled trial, and 3) to identify potential programme adaptations for improvement.

### The programme

The parenting programme, named ‘the Sinovuyo (‘we have joy’) Teen Programme’ comprised 12 weekly sessions, each lasting 2–2.5 h (with some sessions starting late when roads flooded). Sessions were conducted in local church halls or, where no room large enough was available, under a tree. For eight sessions caregivers and adolescents attended jointly, and in four sessions they attended in separate caregiver and adolescent groups. The programme followed core principles of evidence-based parenting programmes, including collaborative (rather than didactic) problem-solving, home practice and discussion, and skills-based active participation. Sessions, outlined in Table 1, included praising each other, managing anger and stress, joint problem-solving, non-violent discipline, rules and routines, keeping adolescents safe in the community, and responding to crises. Key differences from HIC-based programmes included use of role-plays (instead of videos), simplified session content, adding mindfulness-based physical exercises for stress reduction, and additions of culturally-relevant songs and games. A peer-support system of ‘Sinovuyo buddies’ was introduced to help participants between sessions, as low literacy levels limited the use of written materials. A simple lunch was included as many participants found concentration difficult due to hunger. At the request of participants (but not included in the manual) each session started with a prayer. The manual is available free on request.

**Table 1:** Description of Sinovuyo Teen Programme sessions

Session	Configuration	Goal
1: Introducing the programme & defining participant goals	Joint^a^	Introduce the programme and establish common ground rules and goals.
2: Building a positive relationship through spending time together	Joint	Building a positive relationship while spending time with each other.
3: Praising each other	Joint	Understand the benefits of praise and practicing ways of praising.
4: Talking about emotions	Separate^b^	Learn to identify, name, and discuss emotions.
5: What do we do when we are angry?	Separate	Managing anger and solving problems.
6: Problem solving: Putting out the fire	Joint	Learn the techniques of problem solving.
7: Dealing with problems without conflicts I	Separate	Identify problem behaviours and focus instead on the behaviours you want.
8: Dealing with problems without conflicts II	Separate	Learn relevant and non-harmful alternatives to violent discipline.
9: Establishing rules and routines	Joint	Establishing family rules and routines.
10: Keeping safe in the community	Joint	Make a plan to keep adolescents safe in the community.
11: Responding to crisis	Joint	Combine active listening, anger reduction and problem-solving to help caregivers and adolescents respond to abuse and crisis.
12: Widening the circle of support	Joint	Plan how to move on from here and identify support structures that can help us.

^a^In joint sessions, caregivers and adolescents participated together

^b^In separate sessions, caregivers and adolescents participated in separate sessions

## Methods

### Participants

Participants (*n* = 230) were 115 adolescent-caregiver dyads (adolescents aged 10–17), living in six deprived rural and peri-urban communities. Two thirds of participating families were referred by NGOs, schools, clinics, chieftains and social workers, based on family conflict or challenges in dealing with adolescents. The remainder of families were approached door-to-door in the same communities. No eligibility exclusions were made regarding any factors such as parental or adolescent literacy, mental or physical health or domestic violence. Caregivers and adolescents were invited to participate in a 12-session, weekly parenting support programme. Following other parenting programmes and to avoid stigmatisation, the programme was presented in the community as aimed at reducing parenting stress and family conflict [17].

### Procedures

Pre-post tests with standardized paper questionnaires were completed by adolescents and primary caregivers, prior to the programme and two to six weeks after the final session. A two-stage baseline interview process was used: the first interview included less sensitive items and built trust between participants and interviewers, and the second interview included items such as abuse and harsh parenting. Ethical protocols were approved by the Universities of Cape Town (Department of Psychology Ethics Review Committee PSY2014-001) and Oxford (Inter-Divisional Research Ethics Committee SSD/CUREC2/11-40), the European Research Council (Executive Agency ERC-2012-StG 313421-PACCASA) and provincial Departments of Social Development and Basic Education. Written informed consent was obtained from all participants and, given low literacy levels, consent procedures were also read aloud. Interviewers were trained in working with vulnerable families. No incentives were offered, but each family received a ‘thank-you pack’ chosen by our Teen Advisory Group of South African adolescents. These contained a certificate of participation, a snack and a toothbrush. Confidentiality was maintained, except if participants were at risk of significant harm or requested assistance. If participants reported severe abuse, rape, or other significant harm, immediate referrals were made to child protection, health and HIV/AIDS services, with follow-up support (*n* = 8). All research materials were translated into Xhosa and checked by back-translation. Additionally, each session was observed by a research team member in order to note participant responses, intervention fidelity and collaborative approaches.

### Measures

Primary outcome measures were completed independently by adolescents and caregivers, in order to compare perspectives. *Abuse of adolescents within the home (physical abuse/violent discipline, emotional abuse and neglect)* were measured using the child and parent version of the International Society for Prevention of Child Abuse and Neglect Child Abuse Screening Tool (ICAST-Child, 18 items; and ICAST-Parent, 22 items) [18, 19]. This measure was selected based on its availability free of charge, the range of items covered and its successful use in two previous intervention studies in sub-Saharan Africa [20, 21]. Reliability was α = 0.85 caregiver report and α = 0.79 adolescent report. *Parenting:* Positive and involved parenting (16 items), poor monitoring and inconsistent discipline (16 items) were measured using child and parent subscales of the Alabama Parenting Questionnaire [22], used in South Africa [23] (scale reliability α = 0.69 parent and adolescent report). *Adolescent behaviour problems* were measured using the parent and child subscales for rule-breaking and aggressive behaviour (35 items) of the Child Behaviour Checklist [24], with established validity in multiple countries [24, 25]. Reliability was α = 0.88 caregiver report and α = 0.71 adolescent report.

#### Secondary/linked outcomes

*Caregiver depression* was measured using the Centre for Epidemiologic Studies Depression Scale (20-items), α = 0.86 [26]. *Adolescent depression* used the Child Depression Inventory (CDI) short form [27] (10 items), α = 0.58. *Parenting stress* was measured using the Parental Stress Scale (18 items), α = 0.80 [28]. *Substance use* was measured for adolescents using two items from the WHO Global School-based Health Survey [29] and for caregivers using the WHO ‘ASSIST’ scale (8 items, α = 0.71) [30]. *Social Support* was measured for caregivers using the MOSS Social Support Survey [31] (19 items, α = 0.95) and for adolescents using the SAHA Social Support Scale [32], to identify caregiver support (α = 0.86). *Sexual abuse* (primarily occurring outside the home [33]) was measured using the ICAST-C (α = 0.80) and ICAST-P subscales (α =0 .42). *Witnessing violence in the community* is often increased amongst adolescents who do not want to spend time at home, or where caregivers have low control over evening or alcohol-related adolescent activities, and was measured using four items from the child version of the standardized *Exposure to Violence Scale* [34].

#### Socio-demographic factors and child abuse risk factors

Child and caregiver age, gender, language, household type and structure and household employment used items adapted from the South African Census [35]. Poverty was assessed using four items from the SA National Food Consumption Survey [36], and access to the top eight socially-perceived necessities for children endorsed in the SA Social Attitudes Survey 2006 [37]. Family AIDS and AIDS-orphanhood was measured using the Verbal Autopsy questionnaire [38] (18 items), validated with sensitivity of 83 % and specificity of 75 % in South Africa [39]. *AIDS-related stigma* was measured using the Stigma-by-Association Scale [40], validated for South Africa [41]. *Caregiver’s history of maltreatment during childhood* was assessed using an adapted version of the ISPCAN Child Abuse Screening Tool-Retrospective (ICAST-R) [42] (15 items). *Caregiver exposure to* intimate partner violence was measured using the revised Conflict Tactics Scale (10 items, α = 0.50) [43].

### Analyses

Analyses were conducted using SPSS 21.0. To examine associations of the intervention on adolescents and caregivers, paired t-tests comparing baseline and post-test scores were employed [44]. *Missing data:* All participants were sought for interview at post-test, regardless of attendance levels. Of an initially-approached 119 dyads, four dyads dropped out of the study prior to baseline data collection and so lacked any data for inclusion in analyses. All remaining participants (115 dyads) were included using an intention-to-treat approach, whereby outcome analyses include all participants present at the time of pre-intervention regardless of extent of programme attendance or completion. Twelve participants were not interviewed at post-test: 5 had moved to different provinces and could not be located, 4 dropped out of the programme, 2 refused post-test interview, and one caregiver had died during the intervention. These participants’ baseline scores were carried forward to post-test, which provides a conservative estimate of the intervention effect when outcomes are not expected to spontaneously deteriorate, as in the current study [45].

For the vast majority of variables missing data was less than 5 %. When scale items were combined, a total of two out of 23 scales had missing data >15 %: (a) baseline child abuse using caregiver report and (b) baseline problem behaviour using adolescent report. For these, all scale items were imputed. Otherwise, whole scales were imputed separately for adolescent- and caregiver-reported data [46]. Multiple imputation was conducted in SPSS [47], using fully conditional specification with predictive mean matching and included all baseline and post-test scales to improve estimation [48]. As recommended by Graham [49], analyses used the pooled results from 20 imputed datasets. To test the robustness of results to the missing data strategy, a complete-case analysis was also conducted (i.e., including only those participants with complete data). There were no differences in statistical significance or outcomes compared to the imputed analyses, nevertheless the data from the imputed analyses are discussed below; and both are included in Tables 4 and 5.

## Results

### Participant socio-demographics and risk factors for abuse (Table 2)

**Table 2:** Socio-demographic and child abuse risk factors among sample at pre-test

Soico-demographic and risk factors	Adolescents (*N* = 119)	Caregivers (*N* = 119)
Female	58 (49.6 %)	112 (94.1 %)
Age	13.9 (2.3)	47.8 (13.6)
Marital status:		
Single	-	53 (44.5 %)
Married	-	36 (30.3 %)
Widowed	-	20 (16.8 %)
Xhosa spoken at home	115 (99.1 %)	118 (99.2 %)
House type:		
Brick or concrete	78 (66.7 %)	71 (59.7 %)
Traditional materials	23 (19.7 %)	31 (26.1 %)
Shack	13 (11.1 %)	17 (14.3 %)
Orphanhood		35 (29.4 %)
Caregiver child relationship:		
Biological parents	-	62 (52.1 %)
Grandparents	-	30 (25.2 %)
Aunts or uncles	-	22 (18.5 %)
Siblings	-	2 (1.7 %)
Foster parents	-	2 (1.7 %)
Caregiver employed	-	13 (10.9 %)
Someone else in household is employed	-	23 (19.8 %)
4 or more necessities missing	24 (20.2 %)	35 (29.4 %)
Days per week without food	1.0 (2.6)	1.8 (2.6)
Childhood experience of maltreatment	-	58 (48.7 %)
Experiencing intimate partner violence	-	59 (49.6 %)
Poor health in past month	48 (40.3 %)	71 (59.7 %)
Difficulty doing household tasks	-	57 (47.9 %)
HIV-test positive or AIDS-unwell	33 (31.4 %)	50 (41.3 %)

Data are N (%) or mean (standard deviation)

Of 119 families approached, 115 agreed to take part in the study. Fifty-two percent of the primary caregivers were biological parents of the adolescent, 25 % grandparents or great-grandparents, 19 % aunts or uncles, 2 % siblings and 2 % unrelated foster parents. 94 % of caregivers were female, 45 % were schooled to primary level or less, and they reported an average of 1.8 days insufficient food in the past week. Forty-one percent of caregivers and 31 % of adolescents reported HIV+ diagnoses or more than three AIDS-defining opportunistic illnesses. Forty-nine percent of caregivers reported a history of violent discipline in their childhoods, and 50 % were currently experiencing intimate partner violence. Adolescents were 49 % female, 29 % orphaned, and their average age was 14 (SD 2.3).

### Attendance and acceptability

Programme completion was 98.3 %. Attendance at workshops was 62.1 % for adolescents and 53.2 % for caregivers, limited by frequent illness and funerals, characteristic of very high HIV-prevalence areas. When participants were unable to attend, often due to illness or funerals, they were visited at home after the session and asked if they would like to receive a ‘khaya (home) catch-up’ of the session. Acceptance of home catch-ups was very high, thus overall programme receipt was 84.2 % for adolescents and 82.2 % for caregivers (see Table 3). Observations in each session reported that the NGO community workers who were conducting the programme for the first time delivered the programme with high levels of implementation fidelity. Despite challenges in training with collaborative approaches, which were very unfamiliar in a context where education is traditionally didactic and punitive, observations reported that staff conveyed principles of positive reinforcement and collaborative learning [50, 51].

**Table 3:** Summary of programme attendance and receipt

Programme attendance and receipt	Caregiver	Adolescent
Sessions attended in person, out of 12 (mean, %)	6.4 (53.2 %)	7.5 (62.1 %)
Sessions received including home visit catch-ups (mean, %)	9.9 (82.2 %)	10.1 (84.2 %)
Participants receiving all 12 sessions	88 (76.5 %)	91 (79.1 %)

### Primary outcomes

Means and standard errors for all outcome variables at pre- and post-test as well as the t- and p-values for each pre-post comparison are given in Table 4 for adolescents and Table 5 for caregivers. *Abuse of adolescents within the home (physical, emotional, neglect)* significantly decreased following the intervention (*p* < 0.001 adolescent and caregiver reports), dropping from an average score of 4.33 (SE 0.57) to 1.33 (SE 0.27) for adolescents and an average score of 11.32 (SE 0.84) to 1.68 (SE 0.36) for caregivers. Proportions of adolescents reporting within-home abuse were 63.0 % at pre-test, and 29.5 % at post-test, and proportions of caregivers reporting within-home abuse were 75.5 % at pre-test and 36.5 % at post-test.

**Table 4:** Adolescent-report outcomes at pre- and post-test for complete cases and pooled imputed datasets

Outcome	Complete cases^a^				Multiple imputation (*N* = 115)		
	Pre-test	Post-test	t (p)	N	Pre-test	Post-test	t (p)
**Physical abuse, emotional abuse, and neglect, /60** ^**b**^	**4.16 (0.59)**	**1.26 (0.28)**	**4.68 (<0.001)**	**101**	**4.33 (0.57)**	**1.33 (0.27)**	**5.05 (<0.001)**
Sexual abuse, /7	0.16 (0.07)	0.06 (0.04)	1.69 (0.09)	107	0.19 (0.07)	0.08 (0.04)	1.84 (0.07)
**Adolescent behavioural problems, /66**	**8.50 (0.64)**	**5.95 (0.59)**	**3.64 (0.001)**	**76**	**8.64 (0.49)**	**6.40 (0.53)**	**3.77 (<0.001)**
**Positive and involved parenting, /64**	**49.67 (1.00)**	**51.80 (0.95)**	**2.02 (0.05)**	**99**	**48.71 (1.07)**	**51.62 (0.91)**	**2.46 (0.01)**
**Poor monitoring, inconsistent discipline, /76**	**19.28 (1.06)**	**14.61 (0.93)**	**3.63 (<0.001)**	**97**	**19.64 (1.01)**	**15.52 (0.90)**	**3.39 (0.001)**
**Corporal punishment, /12**	**2.66 (0.26)**	**1.28 (0.20)**	**4.35 (<0.001)**	**109**	**2.73 (0.26)**	**1.37 (0.22)**	**4.45 (<0.001)**
**Depression, /20**	**3.02 (0.27)**	**1.37 (0.22)**	**5.12 (<0.001)**	**97**	**3.17 (0.27)**	**1.41 (0.22)**	**5.65 (<0.001)**
Alcohol consumption, /3	0.07 (0.03)	0.11 (0.03)	1.00 (0.32)	111	0.08 (0.03)	0.11 (0.03)	1.00 (0.32)
Drug use, /3	0.11 (0.05)	0.09 (0.04)	0.31 (0.76)	111	0.10 (0.04)	0.09 (0.04)	0.31 (0.76)
**Social support from caregivers, /38**	**34.48 (0.51)**	**36.87 (0.20)**	**4.72 (<0.001)**	**100**	**34.26 (0.51)**	**36.71 (0.21)**	**4.87 (<0.001)**
**Adolescents witnessing violence, /28**	**3.17 (0.41)**	**1.48 (0.31)**	**3.61 (<0.001)**	**111**	**3.25 (0.41)**	**1.54 (0.34)**	**3.58 (<0.001)**

Data are mean (standard error). Statistically significant differences (*p* < 0.05) between pre- and post-test are bolded

^a^N for complete cases varies based on scale, ranging from 50 to 110 caregivers

^b^Value indicates the maximum total score

x

**Table 5:** Caregiver-report outcomes at pre- and post-test for complete cases and pooled imputed datasets

Outcome	Complete cases^a^				Multiple imputation (*N* = 115)		
	Pre-test	Post-test	t (p)	N	Pre-test	Post-test	t (p)
**Physical abuse, emotional abuse, and neglect, /79** ^**b**^	**6.88 (1.19)**	**1.70 (0.45)**	**4.20 (<0.001)**	**50**	**11.32 (0.84)**	**1.68 (0.36)**	**11.24 (<0.001)**
Sexual abuse, /2	0.06 (0.03)	0.01 (0.01)	1.68 (0.10)	107	0.06 (0.03)	0.01 (0.01)	1.92 (0.06)
**Positive discipline, /8**	**5.23 (0.38)**	**2.59 (0.30)**	**5.80 (<0.001)**	**75**	**5.44 (0.28)**	**2.59 (0.24)**	**8.45 (<0.001)**
**Adolescent behavioural problems, /74**	**15.76 (0.93)**	**11.84 (0.73)**	**4.46 (<0.001)**	**99**	**16.16 (0.95)**	**12.14 (0.75)**	**4.37 (<0.001)**
**Positive and involved parenting, /64**	**48.55 (1.07)**	**53.91 (0.82)**	**4.49 (<0.001)**	**99**	**49.23 (0.98)**	**53.83 (0.81)**	**4.04 (<0.001)**
**Poor monitoring and inconsistent discipline, /64**	**24.18 (1.32)**	**16.67 (1.01)**	**5.21 (<0.001)**	**96**	**24.36 (1.21)**	**16.87 (1.06)**	**5.55 (<0.001)**
**Depression, /80**	**23.23 (1.56)**	**14.06 (1.31)**	**5.82 (<0.001)**	**86**	**23.43 (1.42)**	**14.29 (1.27)**	**6.24 (<0.001)**
**Substance use, /4**	**0.75 (0.10)**	**0.40 (0.09)**	**3.12 (0.002)**	**110**	**0.74 (0.10)**	**0.40 (0.09)**	**3.17 (0.002)**
**Parenting stress, /72**	**26.04 (1.00)**	**20.87 (0.77)**	**4.54 (<0.001)**	**100**	**26.43 (0.94)**	**21.46 (0.79)**	**4.54 (<0.001)**
**Social support, /38**	**29.32 (0.95)**	**33.55 (0.76)**	**3.71 (<0.001)**	**106**	**29.29 (0.92)**	**33.56 (0.75)**	**3.82 (<0.001)**
Adolescents witnessing violence, /28	0.66 (0.26)	0.55 (0.28)	0.27 (0.79)	67	1.89 (0.67)	0.75 (0.34)	1.57 (0.12)

Data are mean (standard error). Statistically significant differences (*p* < 0.05) between pre- and post-test are bolded

^a^N for complete cases varies based on scale, ranging from 50 to 110 caregivers

^b^Value indicates the maximum total score

*Positive and involved parenting* showed significant improvement following the intervention, as reported by both adolescents (mean pre-test: 48.71 (SE 1.07), post-test: 51.62 (SE 0.91), *p* = 0.01) and caregivers (pre-test: 49.23 (SE 0.98), post-test: 53.83 (SE 0.81), *p* < 0.001). *Poor monitoring and inconsistent discipline* decreased following the intervention, as reported by adolescents (pre-test: 19.64 (SE 1.01), post-test: 15.52 (SE 0.90), *p* < 0.001), and by caregivers, (pre-test: 24.36 (SE 1.21), post-test: 16.87 (SE 1.06), *p* < 0.001). *Adolescent behavioural problems (delinquency and aggression)* also decreased following the intervention, as reported by adolescents (pre-test: 8.64 (SE 0.49), post-test: 6.40 (SE 0.43), *p* < 0.001) and by caregivers (pre-test: 16.16 (SE 0.95), post-test: 12.14 (SE 0.75), *p* < 0.001).

### Secondary/linked outcomes

Both *adolescent* and *caregiver depression* were significantly reduced following the intervention (adolescents pre-test mean: 3.02 (SE 0.27), post-test 1.37 (SE 0.22), *p* < 0.001); caregivers pre-test: 23.23 (SE 1.42), post-test: 14.06 (SE 1.27), *p* < 0.001). Following the intervention*, caregiver parenting stress* significantly decreased (pre-test: 26.43 (SE 0.94); post-test: 21.46 (SE 0.79), *p* < 0.001) and *caregiver substance use* significantly decreased (pre-test: 0.74 (SE 0.10), post-test 0.40 (SE 0.09), *p* = 0.002). *Adolescent substance use* did not significantly change. There were no significant changes in adolescent or caregiver report of past-month *sexual abuse* of adolescents outside the home, although rates were very low, with a mean of adolescent-reported 0.19 abuse experiences at pre-test and 0.08 abuse experiences at post-test, suggesting that a larger sample is needed for adequate testing of this outcome. Caregivers reported that they were accessing improved *social support* (pre-test: 29.29 (SE 0.92), post-test: 33.56 (SE 0.75), *p* < 0.001), and adolescents reported that they were accessing improved *social support from their caregivers* (pre-test: 34.26 (SE 0.51), post-test: 36.71 (SE 0.21), *p* < 0.001). Adolescents also reported a decrease in *witnessing violence in the community*, reporting a mean 3.25 (SE 0.41) violent experiences at pre-test and 1.54 (SE 0.54), (*p* < 0.001) experiences at post-test. There were no significant changes in caregiver report of adolescents’ witnessing of violence, however most caregivers indicated concern at their own limited knowledge of their adolescents’ exposure to violence in the community.

### Diffusion effects

An additional and unanticipated activity was identified at post-test data collection: extensive diffusion of the programme. In all communities, participants reported that they had actively disseminated programme sessions to other families. This included training extended family, neighbours, and fellow travellers on shared taxi-buses. In three sites, participants had started new ‘Sinovuyo groups’ in local churches. School principals and pastors also reported adapting programme sessions for weekly assemblies and sermons. There was a high level of community support from chieftains and other local leaders. Post-hoc focus group discussions (conducted with UNICEF) with child and youth care workers who implemented the programme reported that they were utilising programme skills within all villages in which they worked, as well as within their own families.

‘We also use the 6 steps of problem solving. My child says that I have changed. When he was doing something wrong I used to shout at him without telling the reasons why it was wrong. Now I sit down with him to solve the problem and explain what the problem is, why it is wrong, and we try to find solutions.’ (Focus Group, November 26, 2014).

Also unanticipated throughout the programme implementation, local government officials regularly visited sessions, questioned participants about their experiences, and developed plans for scale-up if research results remain positive in a forthcoming randomised trial.

## Discussion

This is the first known quantitative study of a child abuse-prevention programme for adolescents in a LMIC, albeit an early stage test of the programme, using a pre-post design without comparison group. Following the intervention, adolescents and caregivers independently reported reductions in child abuse, adolescent problem behaviour, and negative parenting, and improvements in positive and involved parenting, compared to their levels at baseline. Analyses of secondary outcomes showed additional reductions in adolescent and caregiver depression, parenting stress and caregiver substance use as well as improvements in perceived receipt of social support among both adolescents and caregivers. Overall, the study showed no evidence of harmful effects and positive changes on all primary outcomes (8 out of 8) and most secondary outcomes (7 out of 11). Of course it is important to be cautious about pre-post results without a control group, but these findings suggest possible programme benefits in a range of outcomes, and viability of the programme for further testing in a randomised controlled trial.

The study also provides evidence and raises questions about how a parenting support programme may interact with high levels of deprivation in a Southern African context. Challenges to implementation included flooding (making dirt roads in rural areas impassable), outbreaks of xenophobic violence, and tensions surrounding concurrent national elections that resulted in safety concerns for participants, NGO and research staff. It was essential to establish clearly that the programme was not associated with a political party or electioneering, whilst gaining the support and consent of local politicians and leaders – a difficult balance. Participants also reported high levels of poverty, intimate partner violence and HIV/AIDS, and low levels of education and literacy. For example, some children were severely ill throughout much of the programme, and sessions were delivered to them in hospital with visiting families. For other families, very high levels of alcohol or other substance use made engagement difficult and home visits dangerous for staff. Finally, the research was committed to building local capacity rather than bringing expertise from major cities, and as a result training of research and implementation teams was intensive, reflecting the systematic deprivation of educational capacity in ‘homelands’ during apartheid rule. Given these constraints, successful programme completion, high attendance rates and positive outcomes among both caregivers and adolescents were encouraging. These findings can be compared to evidence from high-income countries. For example the Nurse-Family Partnership found that maternal exposure to intimate partner violence diminished overall high programme effectiveness [52], whilst in some trials of Incredible Years parenting intervention in Europe, parents with the highest levels of depression improved most [53].

This pre-post test also provides important lessons for forthcoming stages of a randomised trial and potential scalability. Firstly, participants were included in the programme through a range of community and service referrals and door-to-door visiting – reflecting real-world approaches to identifying families in need in Southern African contexts. This probably resulted in inclusion of families who were less in need of child abuse prevention services, but also certainly resulted in inclusion of families who would often be excluded from initial programme testing, for example those with severe alcohol and drug problems, mental health problems, domestic violence and terminal illness. Second, the findings, measure development and attrition rates of this study have allowed sample size calculations to be made for a forthcoming cluster randomised trial – previously impossible due to lack of any prior studies of prevention of abuse of adolescents in a LMIC. Third, the unanimous support of traditional leaders, provincial and local government, and school principals was essential to implementation feasibility. Although some families initially expressed suspicion, programme acceptability was far higher than expected and staff were approached daily in study sites with requests to join the programme. Indeed, entirely unanticipated, extensive programme diffusion took place within communities, although nothing is known about the quality or fidelity of diffusion activities. Such diffusion has not been recorded in previous literature on parenting programmes. It seems that South African cultural approaches were adapting what had been conceptualized as a small-group intervention into a shared, community-level resource.

The diffusion also had implications for research. It indicated that a planned individually randomised trial would be at high risk for contamination between intervention and control families within the same villages. Consequently, the research design was adjusted to a cluster trial with randomization at a village-level (currently underway, Pan-African Clinical Trials Registry registration number: PACTR201507001119966). It also highlighted the need for qualitative research alongside the trial to better understand the dynamics of a parenting programme in South Africa.

This study raises further questions about the possible mechanisms of change produced by the programme. Skills-building was based on collaborative problem-solving, home practice, and vignettes, following systematic reviews from high-income countries. However, in a complex intervention, we do not know whether these were the vital factors in programme effectiveness. For instance, caregivers reported regular and supportive contact with their ‘Sinovuyo Buddies’ during and beyond the programme, and this ‘unsupervised’ component may have contributed to positive outcomes. Moreover, participants adopted programme songs as reminders of session principles and when staff arrived in villages, children would surround them singing the past week’s song. Thus again, local cultures seemed to have modified, and potentially improved, the programme for their own context.

This study has several limitations. Findings from a pre-post, non-randomised test cannot determine causality and are only indicative of potential programme results. Post-test interviews occurred shortly after intervention and therefore we do not know whether reported changes endured over time, nor the extent to which participants continued to meet with their programme groups or ‘buddies’. It may well be that formal or informal follow-up sessions boost programme effects, but the need for or value of this remains an empirical question in the current context. It would also be valuable in future trials to assess potential impacts on other children and caregivers within study households. Although the study area was chosen for infrastructural challenges and very limited services that may be representative of conditions across Southern Africa, generalizability to other LMIC settings remains unknown and further testing is clearly required. Additionally, there remain questions about how gender, adolescent age and biological/non-biological caregiving relationships may moderate programme effects. Some findings remain unexplained – for example why caregivers reported reductions in substance abuse, but adolescents did not, despite reported reductions in related problems of aggression and delinquency.

Finally, a number of outcomes were analysed, although all were complementary and conceptualised as part of the intervention’s theory of change. Statistical adjustment for multiple testing was consequently not conducted due to concerns of overcorrection and misinterpretation of treatment effects [54], and because tests will be replicated in the forthcoming randomised-controlled trial. Nevertheless, checking with a conservative Bonferroni correction (α = 0.05/19), all effects remained statistically significant except adolescent-reported positive parenting.

Despite these limitations, this study also has notable strengths. External validity is high: local NGO childcare workers (trained by a NGO) ran the programme in low-resource locations without electricity or running water. This pragmatic approach meant that the research team were not able to assure quality or programme fidelity, but reflected real-world conditions of delivery in Southern African contexts. There were no exclusion criteria for participants, reflecting the reality of service provision rather than ideal research conditions. These conditions boost the external validity of findings by reflecting real-world programming in low-resource contexts in Southern Africa. The study also used standardized outcome measures and elicited independent reporting from adolescents and caregivers within each household, bolstering findings that suggest that in low-resource contexts an abuse-prevention programme is feasible and scalable.

## Conclusions

This pre-post test has three key findings. First, it demonstrates feasibility and initial effectiveness of a parenting programme to reduce abuse of adolescents in a low-resource setting. Second, it shows that rigorous parenting research is possible in high deprivation contexts and in close partnership with NGOs and government. Third, it adds to the parenting programme literature as the first study of its kind in a LMIC, taking place in a region with some of the world’s highest rates of child abuse [3]. While this study by no means provides definitive answers, it suggests strong possibilities in a global initiative towards evidence-based solutions.

## Footnotes

1 ‘parent’ and ‘caregiver’ refer in the Southern African context to care provided by any primary caregiver, whether a biological relation or not.
